# Intelligent Detection Method of Defects in High-Rise Building Facades Using Infrared Thermography

**DOI:** 10.3390/s26020694

**Published:** 2026-01-20

**Authors:** Daiming Liu, Yongqiang Jin, Yuan Yang, Zhenyang Xiao, Zeming Zhao, Changling Gao, Dingcheng Zhang

**Affiliations:** 1 School of Mechanical Engineering, Sichuan University, Chengdu 610065, China; 2 China Mcc5 Group Corp., Chengdu 610063, China

**Keywords:** semantic segmentation, DeepLabV3+, YOLOV11, defect detection

## Abstract

High-rise building facades are prone to defects due to prolonged exposure to complex environments. Infrared detection, as a commonly employed method for facade defect inspection, often results in low accuracy owing to abundant interferences and blurred defect boundaries. In this work, an intelligent defect detection method for high-rise building facades is proposed. In the first stage of the proposed method, a segmentation model based on DeepLabV3+ is proposed to remove interferences in infrared images using masks. The model incorporates a Post-Decoder Dual-Branch Boundary Refinement Module, which is subdivided into a boundary feature optimization branch and a boundary-guided attention branch. Sub-pixel-level contour refinement and boundary-adaptive weighting are hence achieved to mitigate edge blurring induced by thermal diffusion and to enhance the perception of slender cracks and cavity edges. A triple constraint mechanism is also introduced, combining cross-entropy, multi-scale Dice, and boundary-aware losses to address class imbalance and enhance segmentation performance for small targets. Furthermore, superpixel linear iterative clustering (SLIC) is utilized to enforce regional consistency, hence improving the smoothness and robustness of predictions. In the second stage of the proposed method, a defect detection model based on YOLOV11 is proposed to process masked infrared images for detecting hollow, seepage, cracks and detachment. This work validates the proposed method using 180 infrared images collected via unmanned aerial vehicles. The experimental results demonstrate that the proposed method achieves a detection precision of 89.7%, an mAP@0.5 of 87.9%, and a 57.8 mAP@50-95. surpassing other algorithms and confirming its effectiveness and superiority.

## 1. Introduction

Rapid global urbanization has led to a proliferation of high-rise structures in modern cities. Due to prolonged exposure to challenging environmental conditions, building facades frequently develop defects—including cracks, hollowing, water seepage, and detachment—which present substantial hazards to public safety. Regular inspections of high-rise building facades can facilitate the timely identification of defects, which is of significant practical importance [1]. Currently, common detection methods are primarily classified into manual inspection and non-destructive testing. Manual inspection methods, such as visual examination, tapping, and pull-off testing, are characterized by operational simplicity. However, detection accuracy relies heavily on personnel experience, and these methods suffer from drawbacks including low efficiency and poor precision [2]. Non-destructive testing methods, such as infrared thermography and ground-penetrating radar, employ advanced techniques to overcome the limitations of manual inspection and have been increasingly applied to the detection of building facade defects [3]. Among these, infrared thermography utilizes differences in temperature field distribution to reveal defects such as hollowing, cracks, and facade detachment [4]. However, infrared images are susceptible to environmental interference, which compromises detection accuracy [5].

Currently, deep learning techniques have been widely applied in the detection of defects in infrared images of building facades. Leveraging their powerful feature learning capabilities, these techniques enable the automatic extraction of representational features for defect identification. This thereby mitigates manual intervention and subjective errors, and consequently enhancing detection efficiency and accuracy [6]. Zheng et al. [7] combined visual Transformers with infrared thermography to achieve high-precision detection of thermal anomalies on building facades; Fu et al. [8] implemented a Swin Transformer-based approach to accurately identify multiple types of surface defects—such as moss growth, efflorescence, contamination, and deterioration—under the complex infrared imaging conditions of heritage buildings; meanwhile, Pan et al. [9] integrated the Mask R-CNN network to enable pixel-level detection of internal defects in FRP–concrete structures using infrared thermography. However, infrared images are highly susceptible to environmental factors such as ambient temperature, sunlight intensity, and background interference, rendering it challenging to distinguish thermal anomaly regions from actual defects. [10] Consequently, direct detection using a single model often intricate architectural environments leads to detection inaccuracies caused by intricate environmental contexts.

The DeepLabV3+ model is an advanced semantic image segmentation architecture developed by the Google research team. It is primarily utilized for pixel-level image segmentation tasks and is characterized by efficient computational cost, high segmentation accuracy, and strong adaptability to multi-scale contexts. The model has been widely applied in fields such as medical image analysis (e.g., chest CT segmentation) [11], autonomous driving (road scene segmentation) [12], fabric defect detection [13], and satellite image processing. DeepLabV3+ model is centered on its Atrous Spatial Pyramid Pooling (ASPP) module, which enables the fusion of multi-scale contextual information and exhibits excellent multi-scale feature extraction capabilities [14]. Previous studies have demonstrated the effectiveness of its structural design across various domains. Wang et al. [15] proposed a lightweight DeepLabV3+ model that achieved high accuracy in high-resolution remote sensing image segmentation. Ali et al. [16] evaluated the generalization capability of DeepLabV3+ for polyp detection and segmentation tasks, showing that the model exhibits strong robustness in local feature extraction. Wang et al. [17] introduced Sh-DeepLabV3+, which effectively enhanced feature extraction performance in the classification of crop residue coverage patterns. Focusing on defect detection, Ye et al. [18] integrated attention mechanisms and dynamic snake convolutions into DeepLabV3+ to improve the automated segmentation of linear road cracks. Furthermore, Liu et al. [19]. developed MFA-DeepLabV3+, a lightweight network designed to address imprecise edge target segmentation through multi-feature fusion and global pyramid attention. Despite these advancements, applying DeepLabV3+ specifically to infrared defect segmentation of building facades remains challenging. Firstly, edge segmentation accuracy in intricate architectural environments remains suboptimal. Secondly, the model’s ability to distinguish actual defects from interferences such as thermal noise is limited, often resulting in mis-segmentation. Finally, conventional loss functions lack targeted optimization for boundary pixels, leading to poor segmentation performance for small-scale defects such as cavities and channel-shaped structural components.

To address the aforementioned limitations, a two-stage intelligent defect detection method tailored for high-rise building facades is proposed in this work to enhance detection accuracy under intricate architectural environments. In the first stage, an enhanced DeepLabV3+ model is employed to accurately detect interferences in infrared images and generate corresponding masks. In the second stage, YOLOV11 is utilized to achieve precise localization and detection of actual defects. To further enhance segmentation performance, a lightweight Post-Decoder Dual-Branch Boundary Refinement Module is integrated after the output of the DeepLabV3+ decoder. This module consists of a boundary feature optimization branch and a boundary-guided attention branch, which operate collaboratively to achieve sub-pixel contour refinement and adaptive boundary enhancement. Through this dual-branch design, the model effectively mitigates edge blurring caused by thermal diffusion and improves its sensitivity to critical structural anomalies such as slender cracks and small cavities. In addition, an edge-weighted training strategy based on boundary-aware loss functions is employed to strengthen the model’s sensitivity to small-scale targets and intricate background-interference boundaries.

The structure of the current work is organized as follows: Section 1 elucidates the improved DeepLabV3+ model and its optimization design. Section 2 presents the overall detection workflow. Section 3 describes the experimental dataset and defect classification. Section 4 analyzes the experimental protocol, hyperparameter settings, environmental configuration, and results. Section 5 summarizes the primary research findings and discusses the contributions of the proposed method.

## 2. Interference Removal Model Based on DeepLabV3+

### 2.1. Methodology Framework: From Baseline DeepLabV3+ to Proposed Enhancements

**Figure 1:**
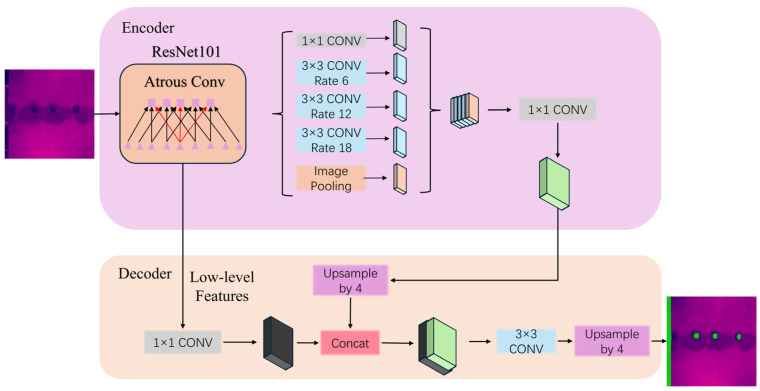
DeepLabV3+ Architecture.

The DeepLabV3+ model inherits and extends an encoder–decoder structure of the DeepLab series, as shown in Figure 1. The encoder section employs a pre-trained backbone network (e.g., DCNN, ResNet, or Xception) in conjunction with atrous separable convolution to extract high-level features. An atrous spatial pyramid pooling (ASPP) module is simultaneously embedded, which processes multi-scale features by paralleling multiple atrous convolution layers with different dilation rates. This design enables the model to capture a broader range of contextual information while preserving computational efficiency. In the decoder section, segmentation accuracy is further enhanced by upsampling low-level features and fusing them with the encoder output.

To further enhance the segmentation capability of the DeepLabV3+ model within intricate infrared imaging environments, two innovative modules are designed to address feature degradation and boundary blurring issues in infrared images: a Post-Decoder Dual-Branch Boundary Refinement Module and a boundary-aware loss function. Additionally, the method of superpixel enhancement is introduced to improve segmentation performance.

### 2.2. Post-Decoder Dual-Branch Boundary Refinement Module

**Figure 2:**
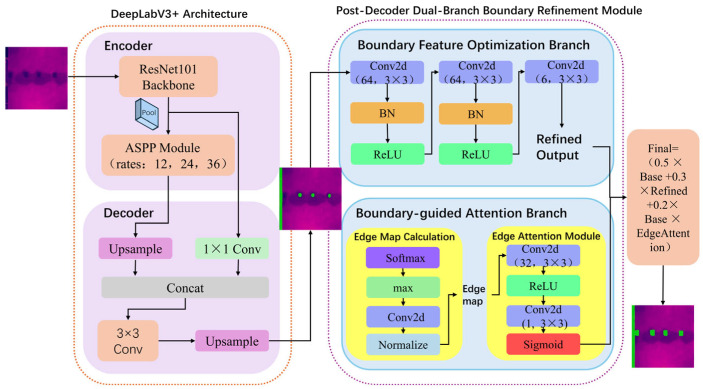
Improved DeepLabV3+ Model Architecture.

To address the infrared image segmentation challenges described in Section 2.1, this study proposes a lightweight Post-Decoder Dual-Branch Boundary Refinement Module, which is integrated as a ‘plug-and-play’ component at the output of the DeepLabV3+ decoder. This allows for fine-grained processing of the preliminary feature maps without modifying the original encoder–decoder architecture. Specifically, the module draws on the core idea of ABANet [20]—utilizing Attention Gates (AG) and hybrid loss to enhance boundary recognition—assimilates its theoretical essence of reinforcing boundary features, and introduces structural improvements for the edge blurring problem in infrared building facade detection tasks. Unlike the original network’s design that embeds AGs within skip connections, this study reconfigures it into a dual-branch module containing the Boundary Feature Optimization Branch and the Boundary-Guided Attention Branch, the structure of which is shown in Figure 2.

#### 2.2.1. Boundary Feature Optimization Branch

**Figure 3:**
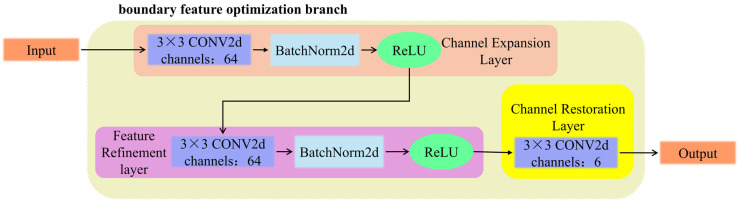
Illustration of the Boundary Feature Optimization Branch.

To address the feature degradation induced by deep convolutional downsampling and the inherent thermal diffusion effect in infrared imagery, the proposed Boundary Feature Optimization Branch provides a solution through a dual mechanism of spatial reconstruction and enhancement. As illustrated in Figure 3, this branch reinforces boundary features through three progressive convolutional operations. First, the Channel Expansion Layer utilizes a 3 × 3 convolution to project input channels into a 64-dimensional space. This dimensionality expansion broadens the parameter representation space, enabling subtle representational features for defect identification—previously smoothed in low-dimensional logits—to be recovered and amplified via rich intermediate representations. Subsequently, the Feature Refinement Layer integrates batch normalization and non-linear activation functions to characterize the topological relationships between local pixels within the high-dimensional feature space. This non-linear mapping mechanism specifically counteracts the pixel-smoothing effect induced by downsampling, thereby reconstructing the boundary sharpness compromised by thermal diffusion. Finally, the Channel Restoration Layer remaps the refined high-dimensional features back to the classification dimensionality. Through residual fusion with the primary path, this layer not only ensures consistency in predictions but also injects enhanced geometric details into the final output, thereby improving the model’s boundary capture capability for weak targets such as cracks.

#### 2.2.2. Boundary-Guided Attention Branch

**Figure 4:**
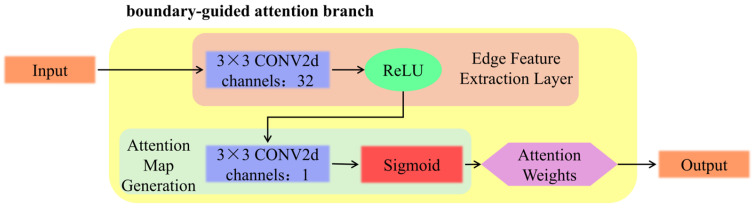
Illustration of Boundary-guided Attention Branch.

The boundary-guided attention branch consists of two components: edge feature extraction and attention map generation (as shown in Figure 4). This branch is specifically designed to counteract the spatial feature degradation caused by multiple downsampling layers in the backbone network, which often results in the loss of slender defect structures and blurred edges. Firstly, the edge detection is performed using a Laplacian convolution kernel based on the maximum class probability map of the initial segmentation result in Equation (1):

(1) $$E=\left|\nabla^{2}\left(P_{base}\right)\right|$$

where E represents the edge feature map, $P_{base}$ denotes the probability map of the initial segmentation result, and $\nabla^{2}$ refers to the discrete Laplacian operator. By operating on the probability map, the module effectively captures the high-frequency uncertainty regions that are most susceptible to degradation during feature decoding.

The resulting edge map is normalized and then fed into a two-layer convolutional network: the first layer employs a 3 × 3 convolution with 32 channels to extract edge features in Equation (2).

(2) F=ReLU(W1⊗E+b1)

where F represents the intermediate feature map; ReLU denotes the activation function; $W_{1}$ is the weight of the first convolutional layer; ⊗ signifies the convolution operation; and $b_{1}$ is the convolution bias term. This stage acts as a high-dimensional feature reconstruction process, transforming sparse edge responses into dense semantic representations to recover the structural details lost in the deep layers of the encoder.

The second layer fuses features through a 3 × 3 convolution and generates a spatial attention map in Equation (3):

(3) $$A=\sigma \left(W_{2}⊗F+b_{2}\right)$$

where A represents the spatial attention map; σ denotes the Sigmoid activation function; $W_{2}$ is the weight of the second convolutional layer; ⊗ signifies the convolution operation; and $b_{2}$ is the convolution bias term. The use of a sigmoid-activated spatial attention map allows the model to perform “feature re-focusing,” assigning higher weights to degraded boundary pixels while suppressing non-structural background noise.

Ultimately, the segmentation performance is enhanced through an attention modulation mechanism in Equation (4):

(4) $$Y_{final}=Y_{base}+\lambda \left(Y_{base}⊙A\right)$$

where $Y_{final}$ represents the final output; $Y_{base}$ denotes the initial segmentation result; λ is the modulation coefficient; and ⊙ signifies element-wise multiplication. Through this residual-like attention coupling, the module re-injects high-frequency geometric information back into the primary feature stream. This mechanism ensures that even if certain features were degraded during the ASPP or upsampling stages, they are adaptively compensated and sharpened before the final prediction.

### 2.3. Improvement of Loss Function

Traditional loss functions typically employ either cross-entropy loss or multi-scale Dice loss independently. However, a single loss function often fails to capture the multi-dimensional complexity of infrared defect segmentation, particularly under conditions of extreme class imbalance and blurred boundaries characteristic of building facades. To address issues such as class imbalance and limited segmentation capability for small targets, a boundary-aware loss $L_{Boundary}$ is introduced and combined with the aforementioned losses to form a triple constraint mechanism in Equation (5):

(5) $$L_{Total}=L_{CE}+L_{Dice}+\lambda \cdot L_{Boundary}$$

where $L_{CE}$ represents the cross−entropy loss; λ is the weighting coefficient; and $L_{Boundary}$ is the weighting coefficient;

The introduction of the boundary-aware loss is fundamentally rooted in the “thermal diffusion effect” inherent in infrared thermography, which results in blurred defect edges and low-contrast transition zones. Conventional loss functions tend to “average out” the contributions of these boundary pixels, often leading to the omission of slender cracks or the mis-segmentation of cavity outlines. To address this, the boundary-aware loss ($L_{Boundary}$) is formulated through two key steps: boundary extraction and loss reweighting.

(1) Boundary Extraction: First, to isolate structural discontinuities, we apply a specific 3 × 3 Laplacian convolution kernel $K_{Lap}$ to the one-hot encoded ground-truth map Y. As implemented in our model, the kernel is defined as:

(6) $$K_{Lap}=\left[\begin{array}{ccc} -1 & -1 & -1\\ -1 & 8 & -1\\ -1 & -1 & -1 \end{array}\right]$$

The convolution response indicates the gradient magnitude at each pixel. To obtain a clear semantic boundary mask M, we take the absolute value of this response and apply a binary threshold of 0.5:

(7) $$M_{i}=1(|{\left(Y*K\_\{Lap\}\right)}_{i}|>0.5)$$

where i indexes the pixel position, and 1(⋅) is the indicator function. Pixels with $M_{i}=1$ represent the precise boundary regions requiring enhanced supervision.

(2) Loss Reweighting: Following the acquisition of the boundary mask M, the loss calculation is performed. Instead of treating all pixels equally, $L_{Boundary}$ acts as a masked cross-entropy loss that selectively amplifies errors within the identified boundary regions:

(8) $$L_{Boundary}=-\frac{1}{N}\sum_{i=1}^{N}\;M_{i}\sum_{c=1}^{K}\;y_{i,c}\log\left(p_{i,c}\right)$$

Here, N is the total pixel count, K is the number of classes, $y_{i,c}$ is the ground truth, and $p_{i,c}$ is the predicted probability. By multiplying the base cross-entropy loss with the binary mask $M_{i}$, gradient signals from boundary pixels are effectively preserved and strengthened during backpropagation, compelling the model to focus explicitly on refining segmentation contours.

### 2.4. Superpixel Segmentation Augmentation

To address the prevalent material texture interference in infrared images of building facades, baseline methods are susceptible to local noise during pixel-level segmentation, resulting in irregular defect boundaries and misclassification of small-area defects. This limitation is primarily attributed to thermal diffusion and material heterogeneity in infrared thermography, which weaken spatial coherence and reduce the effectiveness of purely pixel-wise supervision. To overcome this issue, superpixel segmentation is introduced as an auxiliary structural prior during training to enforce regional consistency. Superpixels group spatially adjacent pixels with similar visual and thermal characteristics into perceptually homogeneous regions, providing a mid-level representation between pixels and objects. Among existing superpixel methods, Simple Linear Iterative Clustering (SLIC) is a widely adopted algorithm that efficiently generates compact and approximately uniform regions by jointly considering color similarity and spatial proximity [21]. Owing to its simplicity, computational efficiency, and robustness, SLIC is particularly suitable for large-scale infrared images with weak texture cues. In this work, SLIC is not employed as a post-processing step but is integrated into the training process as a region-consistency constraint: superpixel regions are generated from input infrared images to construct auxiliary supervision labels, encouraging consistent predictions within each region. This design effectively suppresses isolated noisy responses and mitigates texture-induced pseudo-edges and fragmented predictions in intricate infrared scenes.

In the implementation, the Simple Linear Iterative Clustering (SLIC) algorithm is employed to generate superpixel regions. The compactness parameter is set to 60 to balance spatial proximity. The number of superpixels is adjusted to 300 based on the input image dimensions, ensuring that each superpixel covers an average of 500–800 pixels. A minimum pixel threshold of 20 is introduced to filter out invalid small regions, hence capturing local details while avoiding over-segmentation.

## 3. Two-Stage Process for Building Facade Defect Detection

**Figure 5:**
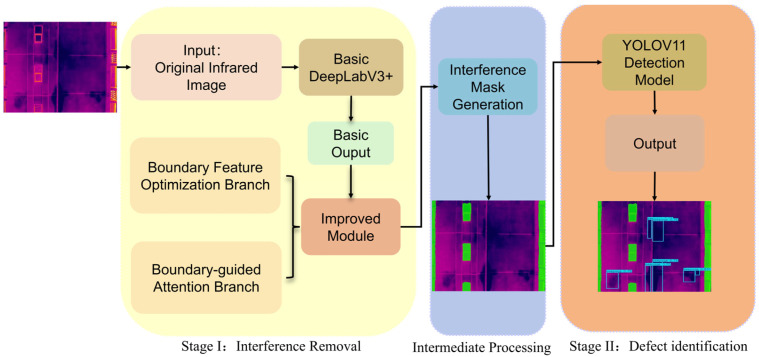
Overall Workflow of Defect Detection.

To achieve high-accuracy detection of façade defects in infrared thermography for high-rise buildings, this work proposes a two-stage intelligent detection framework. The framework systematically addresses intricate architectural environments interference and blurred defect boundaries by integrating an improved DeepLabV3+ segmentation model with the YOLOV11 object detection network. The overall detection workflow, as shown in Figure 5, comprises two stages: interference suppression and defect detection. These two stages are intrinsically linked, enabling a synergistic enhancement of the overall detection performance.

In the first stage, an improved DeepLabV3+ semantic segmentation model is utilized to segment the interference regions and produce the corresponding binary masks, thereby mitigating the influence of thermal anomalies on the subsequent detection process. Based on the mask constraints generated in the first stage, the second stage is carried out using the YOLOV11 model, through which defect detection is performed on the remaining regions.

## 4. Introduction to the Experimental Dataset

### 4.1. Data Sources and Acquisition Methods

**Figure 6:**
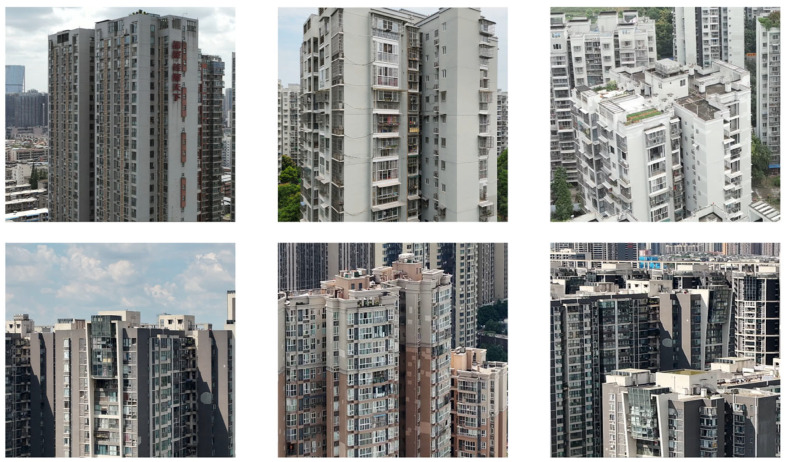
Data Acquisition Building Examples.

The dataset utilized in this work was collected from the façades of 50 high-rise buildings located across more than 30 residential communities in the Chenghua and Longquanyi districts of Chengdu city, covering a variety of façade types including tiles, coatings, and cement mortar. To visually illustrate the data acquisition environment, Figure 6 presents an overview of the representative surveyed buildings. Data acquisition was carried out under stable meteorological conditions, with the average ambient temperature maintained above 10 °C. A DJI M350 RTK unmanned aerial vehicle (DJI Innovations, Shenzhen, China) equipped with an infrared thermal imager was employed to perform precise inspection of defective façade areas. The drone was operated according to a predefined flight program and positioning system, with the distance to the façade kept at approximately 10 m to ensure image quality.

### 4.2. Description of Interference and Defect Characteristics

**Figure 7:**
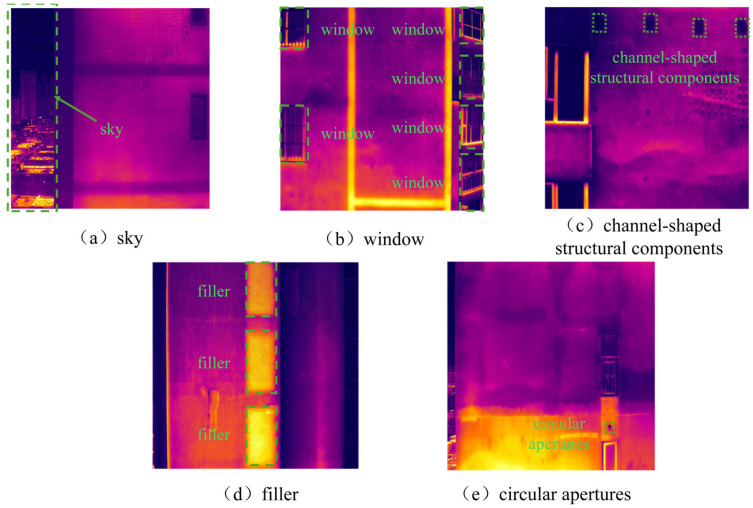
Examples of Five Types of Interference.

The five categories of interference are characterized as follows: sky interference (Figure 7a, green dashed region on the right) is manifested as striped or blocky regions with uneven temperature distribution; window interference (Figure 7b, green dashed region) is exhibited as rectangular or near-rectangular high-intensity thermal anomaly areas; channel-shaped structural component interference (Figure 7c, regularly arranged green dashed rectangles) is typically presented with regular rectangular shapes and periodic arrangement; filler wall interference (Figure 7d, green rectangular region) is characterized by straight boundaries and high regional connectivity; and circular apertures (Figure 7e, small circular holes within the lower-right green rectangle) is generally observed as localized small-scale thermal anomalies with circular morphology and clear boundaries.

**Figure 8:**
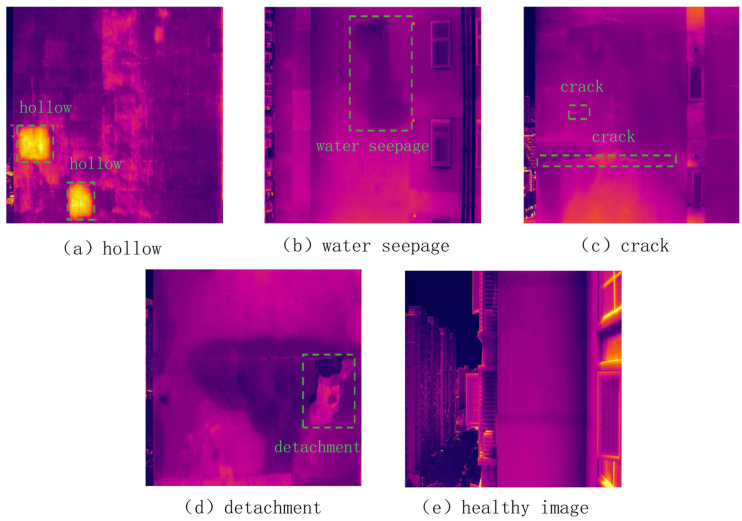
Representative Samples Used For Defect Detection.

The facade defects are primarily classified into four categories: hollowing, water seepage, cracks, and detachment. Specifically: hollowing (shown as the yellow regions within the green frames in Figure 8a) predominantly occurs in building materials such as sandstone and masonry, typically resulting from insufficient tensile strength of the materials; water seepage (indicated by the dark black areas within the green frames in Figure 8b) arises when rainwater penetrates the wall interior through weak points in the facade; cracks (depicted as linear regions within the green frames in Figure 8c) are generated due to uneven foundation settlement, thermal stress, or excessive loading; and detachment (illustrated in the green frame regions in Figure 8d) is caused by loss of adhesion between the finish layer and the substrate or by hollowing, resulting in separation from the main structure under environmental influences. In contrast, Figure 8e illustrates a healthy facade sample (reference image). Unlike the defective regions, it exhibits a relatively uniform thermal distribution without significant temperature anomalies, serving as a baseline for defect identification.

### 4.3. Dataset Construction and Annotation

**Figure 9:**
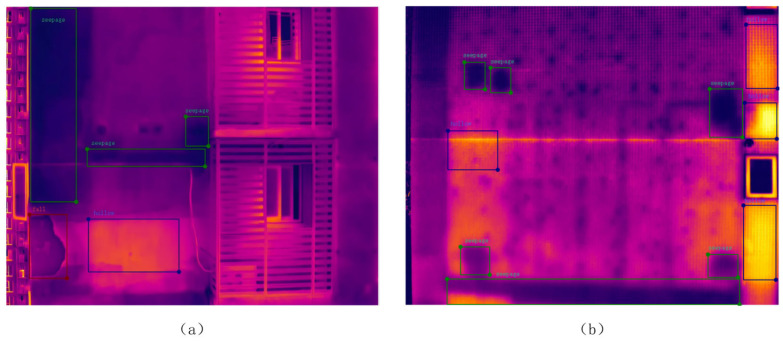
Visualization of Annotated Samples.

To support infrared thermography-based defect detection on high-rise building facades, a comprehensive dataset of 180 images was constructed, in which both interference and defect regions were precisely annotated by experts using LabelMe. The interference instances are categorized into five types—106 sky interferences, 172 window interferences, 52 channel-shaped structural components, 32 filler walls, and 30 circular apertures—to provide reliable support for interference removal, while the defect samples comprise 595 instances across four categories: 416 water seepage defects, 124 hollowing defects, 43 cracks, and 12 detachment defects. To ensure scientific rigor in model training and evaluation, the dataset was randomly partitioned into a training set (125 images) and a test set (55 images) at a 7:3 ratio. The test set comprehensively covers major defect categories with 49 instances (29 water seepage, 16 hollowing, 2 cracks, and 2 detachment defects) and notably includes 43 healthy images; this inclusion aims to rigorously evaluate the model’s specificity, particularly its ability to distinguish between actual defects and normal wall textures. It is worth noting that although the number of training and testing images appears limited, the dataset possesses a high instance density. As illustrated in Figure 9, the majority of these images contain multiple co-occurring defect categories and complex interference types, with a single image often hosting various defects. This ‘dense’ characteristic ensures that the model receives rich and diverse supervisory signals.

## 5. Experimental Methodology

To clearly elucidate the technical details and performance advantages of the proposed two-stage method, a systematic analysis of the improved DeepLabV3+ model is carried out, and then a comprehensive description is provided regarding the design principles of its two modules and their respective contributions to enhancing interference mitigation performance. Subsequently, the contributions of each module are quantitatively assessed through eight sets of interference removal experiments, validating the efficacy of the optimization strategies. Furthermore, six sets of comparative experiments are conducted to comprehensively evaluate the performance differences between the proposed two-stage method and direct detection approaches in façade defect detection from infrared images.

### 5.1. Environment Configuration

The experimental environment was configured on a workstation running Windows 10. In terms of hardware configuration, an Intel(R) Core(TM) i9-14900KF processor is utilized, accompanied by 64 GB of system RAM to meet the computational resource demands of the experiments. For graphics processing, an NVIDIA GeForce RTX 3060 GPU with 12 GB of VRAM is configured, enabling efficient handling of complex graphical and computational tasks. Regarding the software environment, CUDA 12.1 and CUDNN 9.0 GPU acceleration libraries are installed to fully leverage hardware performance and satisfy the requirements of deep learning experiments. The deep learning framework selected is PyTorch 2.4.1, which offers excellent flexibility and robust functionality suitable for this work. Finally, Python 3.12 is adopted as the programming language, providing extensive library support and superior development experience.

### 5.2. Model Evaluation Metrics

To quantitatively evaluate the training performance of the DeepLabV3+ segmentation model and the YOLOV11 detection model, evaluation metrics are employed as direct measures of the models’ segmentation and detection capabilities. In this work, performance is primarily assessed using five key metrics: accuracy (*Acc*), precision (*Pre*), recall (*Rec*), F1 -Score (*F1*) and mean intersection over union (*Miou*). The corresponding calculation methods are presented from Equation (9) to Equation (13).

Accuracy measures the overall correctness of the model’s predictions, reflecting detection reliability. Precision measures the proportion of correctly predicted target objects among all predicted targets, indicating the model’s ability to avoid erroneous identifications. Recall evaluates the model’s ability to identify target objects (e.g., defective regions). Mean Intersection over Union quantifies the agreement between predicted and ground-truth regions in terms of shape, position, and size, serving as the core metric for assessing segmentation precision. The F1-score provides a balanced assessment by combining precision and recall into a single metric, which is particularly crucial for evaluating performance on imbalanced datasets where defects are sparse compared to the background.

(9) $$Acc=\frac{TP+TN}{TP+TN+FP+FN}$$

(10) $$Precision=\frac{TP}{TP+FP}$$

(11) Rec=TPTP+FN

(12) $$Miou=\frac{1}{n+1}\sum_{n=0}^{n}\frac{TP}{TP+FP+FN}$$

(13) $$F1=\frac{2TP}{2TP+FP+FN}$$

where *TP* denotes the number of true positive samples correctly predicted as positive, *FP* represents the number of false positive samples incorrectly predicted as positive, *FN* indicates the number of false negative samples incorrectly predicted as negative, *TN* signifies the number of true negative samples correctly predicted as negative, and n refers to the number of classes.

To further evaluate the performance of the YOLOV11 object detection model in the second stage, two additional metrics *mAP@0.5* and *mAP@50*-*95* are introduced, in addition to the other metrics mentioned above. Their calculations are presented in Equations (14) and (15). The mAP@0.5 and *mAP@50-95* serve as two core metrics in the field of object detection for assessing the comprehensive performance of the model, reflecting its accuracy in target localization and recognition tasks.

(14) $$mAP@0.5=\frac{\sum_{i=1}^{N}AP_{i}\left(IoU_{thresh}=0.5\right)}{N}$$

(15) $$mAP@50-95=\frac{1}{10}\sum_{IoU=0.50}^{0.95}mAP\left(IoU\right)\left(step=0.05\right)$$

### 5.3. Related Hyperparameter Settings

During the training of the DeepLabV3+ segmentation network in the first stage, the input image resolution is treated as a key hyperparameter due to its direct impact on feature representation and computational cost. To evaluate the effect of input scale, multiple image sizes, including 300 × 300, 640 × 640, 800 × 800, and 1280 × 1280, are examined. Smaller resolutions tend to lose fine-grained boundary details of defects, whereas excessively large resolutions significantly increase memory consumption and training instability without providing proportional performance gains. Based on the comparative results, an input resolution of 640 × 640 achieves the best balance between segmentation accuracy, boundary preservation, and computational efficiency, and is therefore adopted for all experiments. Model training is conducted using a mini-batch stochastic gradient descent optimizer. Considering convergence behavior and GPU memory constraints, the batch size is evaluated in the range of [8, 12] and is finally set to 12, while the initial learning rate is examined within [1 × 10^−4^, 5 × 10^−4^] and ultimately fixed at 1 × 10^−4^. A weight decay coefficient of 1 × 10^−4^ is consistently applied to mitigate overfitting while maintaining stable optimization. For the superpixel-assisted supervision module, the Simple Linear Iterative Clustering (SLIC) algorithm is employed to enhance regional consistency in infrared façade images, where the number of superpixels is examined in [100, 300] and finally set to 300, and the compactness parameter is evaluated in [30, 60] and fixed at 60 to better preserve small-scale defect boundaries while enforcing regional regularity. In addition, the boundary loss weight is investigated in [3, 6] and a value of 3 is selected to provide sufficient boundary refinement without overwhelming interior region learning. For noise suppression and region validity control, the Gaussian smoothing parameter σ is preliminarily tested within [0.0, 1.0] and set to 0.5, while the minimum pixel count per superpixel is examined in [10.0, 30.0] and fixed at 20 based on empirical stability and segmentation quality.

In the second stage of defect detection, the YOLOV11 model is employed to detect façade defects using the masks generated in the segmentation stage, with the input image size consistently fixed at 640 × 640 to maintain consistency with the segmentation stage. Training is performed for 300 epochs with a batch size of 12 using the SGD optimizer on a single GPU. To ensure balanced optimization among different detection objectives, the loss weights of the detection head are carefully adjusted. Specifically, the classification loss weight, bounding box regression loss weight, and distribution focal loss weight are examined within the ranges of [0.3, 0.7], [5.0, 10.0], and [1.0, 2.0], respectively. Lower classification loss weights may weaken category discrimination, whereas excessively large values can hinder localization learning. Similarly, insufficient regression loss weighting degrades bounding box accuracy, while overly large values may dominate the optimization process and compromise training stability. The distribution focal loss weight controls the precision of bounding box distribution modeling, where moderate values help balance localization accuracy and convergence robustness. Based on empirical performance and training stability, these loss weights are finally set to 0.5, 7.5, and 1.5, respectively. During inference, the confidence threshold is evaluated within [0.1, 0.3], and the non-maximum suppression IoU threshold is examined in [0.5, 0.7]. The final thresholds are set to 0.2 and 0.6, respectively, to balance missed detections.

To validate the rationality and effectiveness of the selected hyperparameters in the improved DeepLabV3+ model, multiple sets of hyperparameter comparison experiments are conducted, focusing on variations in input image resolution, superpixel count, compactness, and boundary loss weight. The comparative results are summarized in Table 1. The selected configuration yields a favorable overall trade-off across all five evaluation metrics (Accuracy, Precision, Recall, F1, and Miou), demonstrating reliable segmentation performance and effective interference suppression.

### 5.4. Analysis of Challenges in Infrared Interference Removal

**Figure 10:**
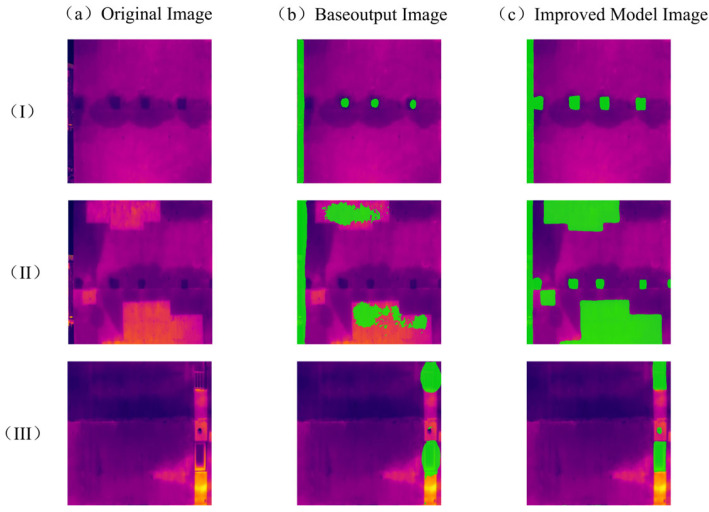
Interference Comparison Results.

To intuitively demonstrate the primary difficulties in infrared segmentation for high-rise building facades and to justify the necessity of the proposed dual-branch refinement module and boundary-aware loss, a comparative analysis between the baseline DeepLabV3+ and our improved model is presented across three representative scenarios (Figure 10). The qualitative results highlight three core challenges that constrain segmentation precision:

(1) Feature Degradation: This challenge manifests as the inability of the model to maintain the structural integrity of small-scale targets. As shown in Figure 10I-a, the channel-shaped structural components possess a distinct rectangular physical profile. However, the baseline DeepLabV3+ model (Figure 10I-b) produces fragmented, dot-like masks, failing to reconstruct the target’s geometric features. This phenomenon stems from the susceptibility of small-scale structural information to loss or degradation during the multi-stage downsampling process within deep convolutional networks. In contrast, the proposed method (Figure 10I-c) successfully produces complete and well-defined rectangular masks for the channel-shaped structural components, thereby ensuring the structural integrity of the target.
(2) Interference from Complex Textures: Infrared imagery is highly sensitive to the inherent textures of building materials [22]. In Figure 10II-a, large-scale interference regions exhibit internal temperature fluctuations due to material non-uniformity. The baseline model is easily confused by these “noisy” thermal textures, leading to the generation of irregular, incomplete masks characterized by internal “hollows”. To address this, our research introduces superpixel consistency constraints and a boundary-guided attention mechanism. These components effectively suppress textural noise and enforce regional consistency within the masks, resulting in full and contiguous segmentation regions, as illustrated in Figure 10II-c.
(3) Boundary Blurring: This challenge originates from the inherent thermal diffusion effect, where heat from high-temperature components bleeds into the cooler surrounding wall areas. In Figure 10III-a, the baseline model (Figure 10III-b) generates rounded and “bloated” masks that significantly exceed the actual physical boundaries of the windows. The proposed method (Figure 10III-c) restores sharp, rectilinear edges through the boundary feature optimization branch, effectively correcting the localization errors induced by thermal diffusion.

### 5.5. Interference Removal Experiments

To thoroughly verify the effectiveness of the improved DeepLabV3+ model in interference removal, nine groups of ablation experiments are designed to analyze the contributions of individual enhancement components. Performance is primarily compared using five metrics: accuracy, precision, recall, F1-Score and mean intersection over union. Training parameters and experimental conditions are kept consistent across all groups.

The specific ablation experiment results are presented in Table 2 (where × indicates that the corresponding module is not used, and √ indicates that the module is included). It can be observed from the results that the enhancement strategies applied to DeepLabV3+ improve model performance to varying degrees. Compared with the baseline model, each enhancement component achieves consistent gains across the four metrics, indicating that all proposed improvements effectively strengthen the model’s capability to remove infrared interference regions. Notably, the most substantial performance improvement is attained when all enhancements are jointly applied: accuracy is increased by 0.1%, precision is improved by 1.7%, recall is improved by 8.3%, the F1 score has increased by 4.8%, and Miou is enhanced by 6.5%.

The results in Table 2 provide empirical evidence of the complementarity and synergistic effects among the modules. By coordinating distinct feature extraction tasks within the challenging infrared environment of building facades, the proposed framework achieves robust performance, enhancing overall segmentation accuracy.

To validate the effectiveness of the first-stage DeepLabV3+ model on infrared images of high-rise buildings, seven comparative experiments were conducted using different models (as shown in Table 3). Training parameters and experimental conditions were kept consistent across all groups. The results indicate that the improved DeepLabV3+ achieved the highest performance in all five evaluation metrics.

### 5.6. Defect Detection Experiments

To verify that defect detection using YOLOV11 [23] after interference removal by the DeepLabV3+ model effectively improves accuracy, recall, mAP@50-95 and mAP@0.5, six groups of defect detection comparison experiments are designed. These experiments are conducted on the original infrared image dataset and the masked image dataset processed by DeepLabV3+ for interference removal, respectively, to evaluate the impact of interference suppression on detection performance. For comparability, training parameters and environmental configurations are kept consistent across all groups. Accuracy, precision, F1, recall, mAP@50-95 and mAP@0.5 are adopted as evaluation metrics to quantify the model’s overall prediction reliability, the accuracy of detected targets, the balanced detection performance under imbalanced data, target detection capability, and comprehensive detection performance, respectively.

#### 5.6.1. Quantitative Comparison of Defect Detection

As shown in Table 4, across different models, performing detection on binary masks after interference removal by DeepLabV3+ generally outperforms direct detection on raw infrared images in all five core metrics, although minor fluctuations are observed in individual models. Specifically, the YOLOV11m model achieves an increase in detection precision from 86.3% to 89.7%, with recall improved from 79.5% to 81.8% and the F1-score rising from 82.8% to 85.6%, and an improvement in mAP@0.5 from 86.7% to 87.9%, while mAP@50–95 increases from 55.6% to 57.8%, demonstrating performance superiority. Similar upward trends are observed in other models, such as SSD [24] and Faster R-CNN [25]. These findings indicate that the preemptive interference suppression process effectively enhances the saliency of representational features for defect identification and reduces the impact of intricate architectural environments on detection, offering advantages over direct detection methods using a single model.

It is worth noting that although the proposed method achieves strong performance in terms of mAP@0.5, its mAP@50–95 is relatively lower due to the intrinsic characteristics of infrared thermography and façade defect targets. Thermal diffusion under infrared imaging often results in blurred and indistinct defect boundaries, introducing uncertainty in both visual appearance and manual annotation, which leads to stricter penalization under higher IoU thresholds (IoU = 0.50–0.95). In addition, defect types such as cracks, water seepage, and detachment exhibit irregular shapes, varying scales, and gradual temperature distributions, making precise bounding box alignment difficult in practice. Nevertheless, under a moderate IoU threshold (IoU = 0.5), the proposed method maintains stable and reliable defect identification.

#### 5.6.2. Per-Class Performance Analysis of Defect Detection

To further investigate the detection performance of the proposed method across different defect categories, a per-class performance analysis is conducted in this subsection. Since YOLOV11m provides a favorable balance between detection accuracy and computational efficiency among the evaluated detectors, it is selected as the representative detection backbone. Accordingly, the per-class detection performance of the baseline YOLOV11m model and the proposed DeepLabV3 + YOLOV11m framework before and after interference removal is compared, as summarized in Table 5 and Table 6, respectively. The evaluation is carried out using precision, recall, F1-score, mAP@0.5, and mAP@50–95 for each defect category.

As shown in Table 5, the baseline YOLOV11m model exhibits notable performance differences across different defect categories, with hollowing and water seepage defects achieving moderate F1-scores but relatively low mAP@50–95 values, indicating greater difficulty in precise localization under complex infrared background conditions, whereas crack and detachment defects attain higher precision and recall. By contrast, the proposed DeepLabV3 + YOLOV11m framework consistently improves detection performance across all defect categories, as summarized in Table 6. In particular, both the F1-score and mAP@50–95 for hollowing and water seepage defects are noticeably enhanced, demonstrating that architectural interference removal effectively increases feature saliency for these hard-to-detect defect types. Meanwhile, crack and detachment defects also benefit from the proposed framework, achieving further improvements in localization accuracy under stricter IoU thresholds.

Overall, this per-class analysis reveals that hollowing and water seepage defects constitute the more challenging categories in infrared façade inspection, primarily due to their diffuse boundaries and gradual temperature variations. The proposed two-stage framework alleviates these challenges by suppressing background interference, thereby improving both detection robustness and class-wise performance consistency.

#### 5.6.3. Qualitative Analysis of Detection Results

**Figure 11:**
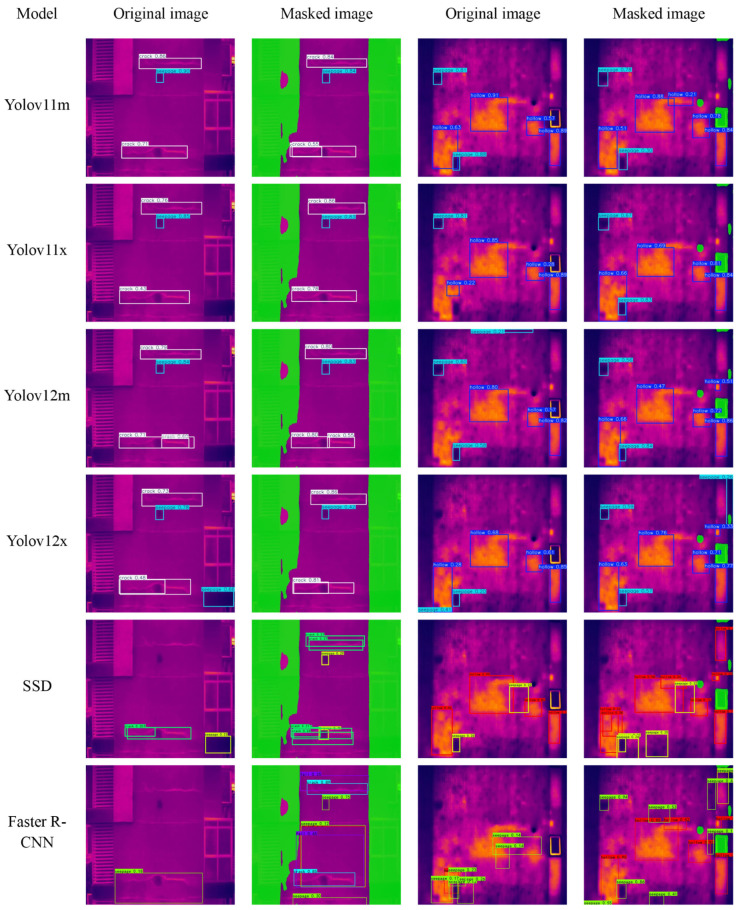
Detection Result.

Further analysis of the detection results (in Figure 11) reveals that when detection is performed directly on raw infrared images, several models exhibit noticeable missed detections. For instance, YOLOV11m, YOLOV11x, and YOLOv12m [26] are unable to the hollowing defect in the lower left corner of the right-side comparison image in rows 1 to 3 of Figure 11; YOLOV12x identifies the lower part of the right-bottom window in the left-side image of the 4th row as a water seepage; while SSD mistakenly identifies the lower part of the right-bottom window in the left-side image of the 5th row in Figure 11 as a water seepage; and Faster R-CNN suffers from multiple missed detections. These missed detections highlight the adverse impact of intricate architectural environments and non-structural interference in infrared images on model judgment. In contrast, when detection is conducted on binary masks after interference removal by DeepLabV3+, the aforementioned erroneous detections are alleviated, and in the current comparison model, there are fewer occurrences of similar erroneous identifications in the limited validation set. Moreover, all models achieve higher detection accuracy and more stable recognition results on the de-noised binary masks, demonstrating particularly superior precision in edge regions and areas with complex textures.

**Figure 12:**
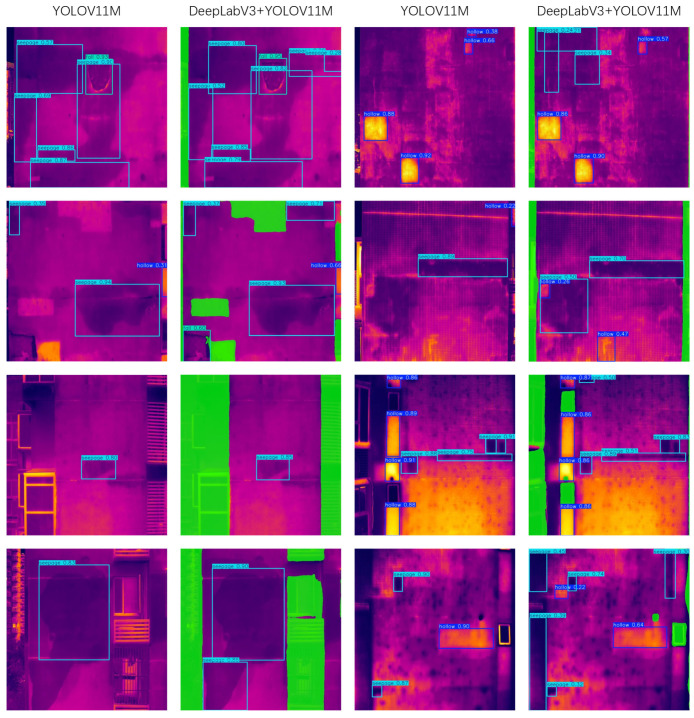
Additional Comparison Result.

To further visually validate the effectiveness of the proposed method, Figure 12 illustrates the comparison of detection results by the YOLOV11M model on raw infrared images versus those processed by the proposed method. As observed in Figure 12, when directly utilizing the baseline YOLOV11M model for identification, multiple significant missed detections of key defects occur due to the influence of intricate architectural environments. In contrast, after applying the proposed interference suppression detection framework, the aforementioned missed defects are successfully detected. This visually demonstrates that the proposed method effectively enhances defect detection capability under complex background scenarios.

In summary, the experimental results comprehensively validate the effectiveness of the proposed two-stage detection method for defect detection in infrared images of building façades. By combining DeepLabV3+ for interference suppression with YOLOV11 for efficient detection, the method mitigates the impact of background noise and enhances the reliability of defect identification in complex scenarios.

## 6. Discussion

In the current study, due to practical constraints related to engineering data acquisition and expert annotation, the corresponding code and dataset cannot be publicly shared at this stage. To further strengthen the credibility of the research conclusions and provide additional evidence for the effectiveness of the proposed method, this section not only elaborates on the contribution and positioning of the proposed framework but also includes a comparative analysis with existing infrared façade defect detection methods.

### 6.1. Analysis of Performance Enhancement Mechanism

The effectiveness of the proposed method stems from the design of the two-stage framework tailored for infrared characteristics. In the first stage, the Post-Decoder Dual-Branch Boundary Refinement Module addresses thermal diffusion and texture interference by reorganizing channel dimensions and employing Laplacian-based attention to reconstruct geometric details. This mechanism compensates for feature degradation, resulting in a segmentation Miou of 78.83% and an F1 score of 87.47%. Based on this foundation, the second stage utilizes a “masking” mechanism to filter out intricate architectural noise—such as windows and sky reflections—that affects direct detection models. Consequently, the framework reduces false detections, increasing the detection precision of YOLOV11m from 86.3% to 89.7%, with the recall reaching 81.8%, mAP@0.5 reaching 87.9%, and mAP@50-95 reaching 57.8%.

### 6.2. Comparison with Existing and Alternative Methods

To further clarify the contribution and positioning of the proposed method, a comparative analysis with existing infrared façade defect detection approaches is presented in this subsection.

Compared with conventional single-stage detection methods that directly perform defect recognition on raw infrared images, the proposed two-stage framework explicitly separates interference suppression from defect detection. As discussed in Section 5.6, direct detection models such as YOLOV11, SSD, and Faster R-CNN are more susceptible to complex architectural interference, which often leads to missed defects. In contrast, by introducing an interference removal stage based on the improved DeepLabV3+ model, the proposed method enhances the saliency of defect-related features, resulting in consistent improvements in precision, recall, and F1-score across different detection backbones.

Furthermore, compared with alternative approaches reported in the recent literature (e.g., Ref. [27]), which rely on direct detection on large-scale infrared datasets, the proposed method places greater emphasis on robustness under complex real-world inspection scenarios. High-rise building façades typically contain windows, sky regions, and structural components with thermal patterns similar to actual defects, posing challenges that are difficult to fully address using direct detection strategies alone. The explicit masking mechanism adopted in this study effectively mitigates the influence of such interference, thereby improving detection stability in densely structured environments. Overall, the proposed method complements existing infrared defect detection approaches by providing an interference-aware detection framework that is suitable for complex architectural environments. This comparative analysis demonstrates that the proposed two-stage strategy offers a practical and effective alternative for real-world high-rise building façade inspections.

### 6.3. Limitations and Future Work

Despite the promising results, this study presents certain limitations that need to be addressed in future work. (1) Dataset Diversity: Although the current dataset covers 50 high-rise buildings, the data were collected primarily under clear weather conditions with temperatures above 10 °C. The model’s robustness under extreme weather conditions (e.g., rain, fog, or extreme cold) has not yet been fully verified. Future research will focus on expanding the dataset to include diverse meteorological scenarios. (2) Defect Quantification: Currently, the system focuses on localization and classification. Incorporating depth estimation or 3D reconstruction to quantify the severity of defects (e.g., depth of hollowing) represents a valuable direction for subsequent research.

## 7. Conclusions

In conclusion, this paper proposes a two-stage detection method based on an improved DeepLabV3+ and YOLOV11. By introducing a dual-branch boundary refinement module and a boundary-aware loss function, the method significantly enhances segmentation accuracy and boundary delineation capability in infrared images. Experimental results validate the high precision of the proposed approach under complex background conditions. The main conclusions are as follows:

(1) The Post-Decoder Dual-Branch Boundary Refinement Module, through the synergistic interaction of the Boundary Feature Optimization Branch and the Boundary-Guided Attention Branch, enhances the segmentation performance of DeepLabV3+ on infrared thermal images. Ablation experiments demonstrate that incorporating this module alone increases Miou by 3.58%, and when combined with other improvements, further elevates the interference segmentation Miou to 78.83%, thereby providing a high-quality mask foundation for subsequent defect detection.
(2) The boundary-aware loss reinforces the model’s ability to discriminate between complex texture interference and true defect boundaries through high-weight supervision of edge pixels, while the superpixel constraint further improves the smoothness of regional predictions. The synergy of both components increases the segmentation recall of the DeepLabV3+ model by 6.27%.
(3) The proposed two-stage defect detection method for building façades reduces interference from elements such as sky and windows. As a result, the YOLOV11m model achieves an mAP@0.5 of 87.9%—the highest among all evaluated methods—and its precision improves from 86.3% to 89.7%, fully validating the effectiveness of the proposed approach.

## Figures and Tables

**Table 1:** Hyperparameter Comparison Experiments.

Learning Rate	Batch	Superpixel Count	Compactness	Boundary Loss Weight	Accuracy (%)	Precision (%)	Recall (%)	Miou (%)	F1 (%)
0.0001	12	300	60	3	99.27	84.58	90.81	78.83	87.47
0.0005	12	300	60	3	99.19	82.68	90.40	76.90	86.18
0.0001	8	300	60	3	99.18	83.23	90.37	77.40	86.52
0.0001	12	100	60	3	99.20	83.38	89.12	76.98	86.10
0.0001	12	300	30	3	99.23	84.52	90.39	78.46	87.30
0.0001	12	300	60	6	99.05	79.84	89.63	73.97	84.14

**Table 2:** Ablation Work Results.

Model	Boundary Feature Optimization Branch	Boundary -Guided Attention Branch	Boundary Loss Function	Superpixel Segmentation	Accuracy (%)	Precision (%)	Recall (%)	Miou (%)	F1 (%)
DeepLabV3+	×	×	×	×	99.16	82.90	82.52	72.34	82.65
	√	×	×	×	99.17	82.72	84.67	73.61	83.63
	×	√	×	×	99.20	84.36	86.71	75.92	85.51
	×	×	√	×	99.16	83.52	83.43	72.68	83.46
	×	×	×	√	99.17	81.59	88.17	74.89	84.63
	√	√	×	×	99.17	82.27	84.54	73.26	83.35
	√	√	√	×	99.18	84.12	89.06	77.25	86.44
	×	√	√	√	99.16	84.05	90.00	77.73	86.82
	√	√	√	√	99.27	84.58	90.81	78.83	87.47

**Table 3:** Comparative Experimental Results on Interference Removal.

Model	Accuracy (%)	Precision (%)	Recall (%)	Miou (%)	F1 (%)
U-net	96.13	76.87	79.63	60.08	78.15
U-net++	98.15	80.44	83.33	70.36	81.84
SAM2	99.19	83.48	84.20	76.68	83.82
Segformer	97.59	82.56	80.99	70.77	81.64
Mask2Former	99.13	84.38	83.67	72.45	84.03
DeepLabV3+	99.16	82.90	82.52	72.34	82.65
Improved DeepLabV3+	99.27	84.58	90.81	78.83	87.47

**Table 4:** Comparative Experiment Results.

Model	Precision (%)	Recall (%)	F1 (%)	mAP@0.5 (%)	mAP@50-95 (%)
YOLOV11m	86.3	79.5	82.8	86.7	55.6
DeepLabV3 + YOLOV11m	89.7	81.8	85.6	87.9	57.8
YOLOV11x	82.6	81.6	82.1	85.8	53.5
DeepLabV3 + YOLOV11x	84.0	82.4	83.2	86.8	54.1
YOLOV12m	67.7	74.5	70.9	80.5	47.1
DeepLabV3 + YOLOV12m	89.0	83.8	86.3	86.2	53.6
YOLOV12x	80.0	80.2	80.1	79.7	45.4
DeepLabV3 + YOLOV12x	70.7	84.8	77.1	83.3	51.5
SSD	86.7	41.6	56.2	67.9	37.9
DeepLabV3 + SSD	88.3	44.3	59.0	63.2	38.7
Faster R-CNN	49.1	66.8	56.6	61.3	37.1
DeepLabV3+ Faster R-CNN	42.3	78.7	55.0	72.9	41.1

**Table 5:** Class-wise detection results of YOLOV11m.

Defect Class	Precision (%)	Recall (%)	F1 (%)	mAP@0.5 (%)	mAP@50-95 (%)	Detection Difficulty
Hollow	83.5	68.8	75.4	76.6	51.9	Hard
Water Seepage	78.9	62.1	69.5	71.1	28.5	Hard
Crack	92.1	95.5	93.8	97.5	55.0	Easy
Detachment	90.6	91.4	91.0	98.5	87.6	Easy

**Table 6:** Class-wise detection results of DeepLabV3 + YOLOV11m.

Defect Class	Precision (%)	Recall (%)	F1 (%)	mAP@0.5 (%)	mAP@50-95 (%)	Detection Difficulty
Hollow	86.3	70.4	77.5	78.8	55.6	Hard
Water Seepage	82.5	62.3	71.0	75.5	29.7	Hard
Crack	96.4	96.2	96.3	98.2	57.4	Easy
Detachment	93.7	98.4	96.0	99.0	88.3	Easy

## Data Availability

The data are currently unavailable, as the project is still under development.
